# Redescription of two species of *Oplitis* Berlese (Acari, Mesostigmata, Oplitidae) from Iran

**DOI:** 10.3897/zookeys.610.9965

**Published:** 2016-08-11

**Authors:** Esmaeil Babaeian, Alireza Saboori, Dariusz J. Gwiazdowicz, Vahid Etemad

**Affiliations:** 1 Department of Plant Protection, Faculty of Agriculture, University of Tehran, Karaj, Iran; 2 Poznan University of Life Sciences, Faculty of Forestry, Wojska Polskiego 71c, 60–625 Poznań, Poland; 3 Department Forestry & Forest Economics, Faculty of Natural Resources, University of Tehran, Karaj, Iran

**Keywords:** Acari, Iran, new species, Parasitiformes, taxonomy, Uropodina

## Abstract

Two new species records of Oplitidae, *Oplitis exopodi* Hunter & Farrier, 1975 and *Oplitis sarcinulus* Hunter & Farrier, 1976 are redescribed based on Iranian specimens from leaf-litter forest in Mazandaran province, northern Iran. A key to the Iranian species of *Oplitis* is presented.

## Introduction

The suborder Uropodina is the most morphologically and ecologically diverse group of mesostigmatic mites. They are free-living or associated with arthropods, mammals, or birds. Worldwide, this suborder comprises approximately 300 genus-group names and 2000 described species (Wiśniewski and Hirschmann 1993, Halliday 2015). *Oplitis* is one of the largest genera of uropodine mites, with nearly 150 described species worldwide. The genus was proposed by Berlese (1884) with *Uropoda paradoxa* Canestrini & Berlese, 1884 as the type species. It has been considered by some authors to be a member of the family Uropodidae (Berlese 1903, 1904, Sellnick 1926, Vitzthum 1942, Hirschmann 1961, Hirschmann and Zirngiebl-Nicol 1964, 1967, Zirngiebl-Nicol 1973a, 1973b, 1973c, Hunter and Farrier 1975, 1976, Hiramatsu 1979, Wiśniewski 1979, Hirschmann 1991), Trachyuropodidae (Karg 1989, Mašán 2001, Kontschán 2013) or Oplitidae (Kontschán 2014, Lopes et al. 2015, Pereira et al. 2016). They are mostly associated with ants but some species were collected from soil and litter. The most recent review of the genus was by Hirschmann (1991), who used a broad concept of the genus and divided *Oplitis* into 16 species-groups, mostly on the basis of features such as presence or absence of a perigenital ring, pre-anal and post anal lines, and the shape of the peritremes and dorsal setae. To date, two species of *Oplitis*, *Oplitis paradoxa* (Canestrini & Berlese, 1884) and *Oplitis iranicus* Kazemi & Kontschán, 2007 have been reported from soil and litter in Iran (Kazemi and Rajaei 2013).

In the present work, two new records of *Oplitis* are reported on the basis of material collected in Mazandaran province, northern Iran, during a survey on Uropodina mites. Also, an identification key to the Iranian species of this genus is presented.

## Material and methods

Mites were extracted from soil detritus and leaf-litter using Berlese-Tullgren funnels, and picked out under a stereomicroscope. After clearing in Nesbitt’s fluid, all specimens were mounted in Faure’s medium on permanent microscope slides. Morphological observations, measurements, and illustrations were made using a BX51 phase contrast Olympus microscope equipped with a drawing tube. Measurements were made from slide-mounted specimens, and are presented as ranges (minimum–maximum) in micrometers (µm). Length of shields and legs were measured along their midlines, and widths at their widest point (if not otherwise specified in the description). Legs I–IV were measured from the bases of coxa to their tips but without the pretarsal ambulacra.

## Taonomy

### Family Oplitidae Hirschmann & Zirngiebl-Nicol, 1964

#### Genus Oplitis

Taxon classificationAnimaliaMesostigmataOplitidae

Berlese, 1884

##### Type species.

*Uropoda paradoxa* Canestrini and Berlese, 1884 by monotypy.

##### Diagnosis.

The most detailed diagnosis of *Oplitis* was provided by Hunter and Farrier (1975, 1976). Species of this genus have dorsal setae spatulate, cuneiform, scimitar-shaped and setiform, but usually have one (sometimes more) basal asymmetric protuberance. Corniculi relatively short; female genital shield free, oval and without setae, located between coxae II–IV; usually with perigenital ring; palp apotele three-tined. Cheliceral digits nearly of similar length; ventral shield with 4–10 pairs of setae; with or without pre-anal line, anal shield bearing two pairs of circumanal setae (*Ad1*, *Ad2*) and a post-anal seta; epistome 3–5 partite and with denticulate margins. Deutosternum moderately wide, smooth and deliminated posteriorly, bearing 3–4 transverse lines of fine denticles between hypostomal setae *h3* and *pc* and behind *pc*.

#### Oplitis exopodi

Taxon classificationAnimaliaMesostigmataOplitidae

Hunter & Farrier, 1975

Figs 1–7 , 10–13

##### Description of females

**(n = 2).** *Idiosoma*. Length 560–570, width 438–458.

**Figures 1–9.**
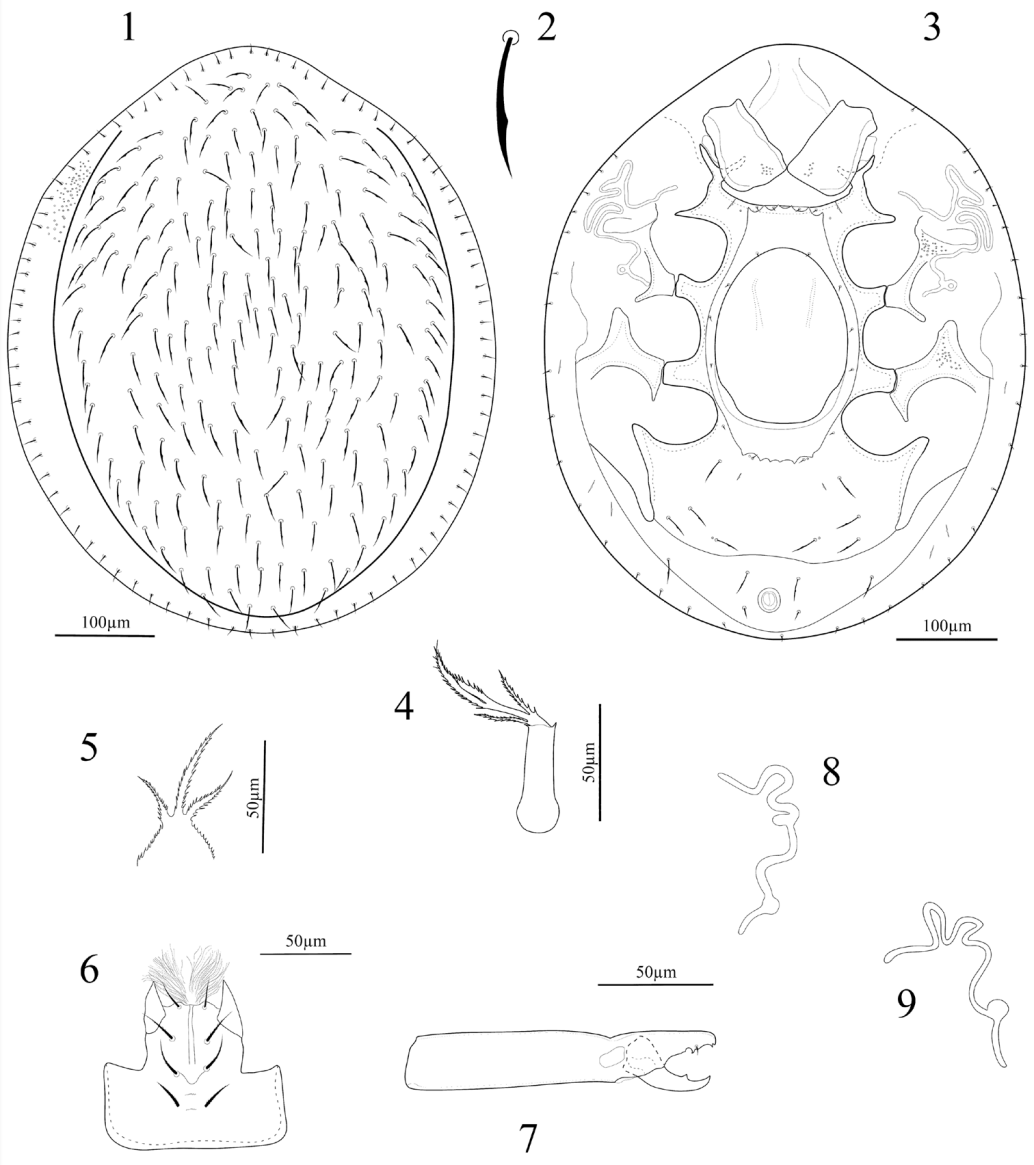
*Oplitis exopodi* (female): **1** Dorsal view of idiosoma **2** Dorsal seta **3** Ventral view of idiosoma **4** Tritosternum **5** Epistome **6** Hypostome **7** Chelicera. *Oplitis pennsylvanica* (female): **8** Peritreme. *Oplitis leonardiana* (female) **9** Peritreme.

*Dorsum* (Fig. 1). Dorsal shield oval, slightly narrowed at both anterior and posterior regions, smooth on whole surface. Dorso-central region with complement of 105 pairs of scimitar-shaped setae (Fig. 2). Marginal shield united anteriorly with dorso-central region, with 42 pairs of smooth and needle-like setae.

*Venter* (Fig. 3). Tritosternal base narrow, c. 43 long, lacinia three-branched and pilose, lateral branches shorter than bifurcate median branch (Fig. 4). Sternal, endopodal and ventral shields smooth, exopodals with small puncta. Genital shield smooth, 157–162 long and 128–133 wide, ratio length/width = 1.21–1.23, situated between coxae II–IV, anterior margin convex and posterior margin straight. Perigenital ring oblong, 235–243 long and 136–143 wide, ratio length/width = 1.69–1.73, extending slightly beyond posterior level of coxae IV, with five crenulations on both anterior and posterior margins; lateral margins smooth and entire, perigenital area with four pairs of very short setae, lateral to genital shield, with two pairs of setae near anterior crenulation, one pair near to posterior crenulation and one pair inserted between coxae IV on inguinal region. Pre-anal line present. Ventral shield smooth, with 4–5 pairs of scimitar-shaped setae, 25–27 in length, *Ad1* scimitar-shaped and 25–26 long, *Ad2* smooth, needle-like and 12–13 long. Peritremes with three subequal convoluted branches. Pedofossae II–IV well developed, pedofossae III foot-shaped and pointed, pedofossae IV narrowing posteriorly and with a rounded tip.

*Gnathosoma* (Figs 5–7). Epistome three-branched and with serrate margins, median branch longer than laterals (Fig. 5). Corniculi short and horn-like; internal malae numerous and brushy, gnathosomal setae *h1* smooth, *h2* with a few barbs, *h3* and *pc* thickened and denticulated (Fig. 6). Cheliceral digits normal for the genus; movable digit 35–38 long, with one subdistal teeth and a terminal hook; fixed digit 47–50 long (from tip to the base of movable digit), with two teeth and a terminal hook; pilus dentilis setiform and minute (Fig. 7). Palptarsus with three-tined apotele.

**Figures 10–13.**
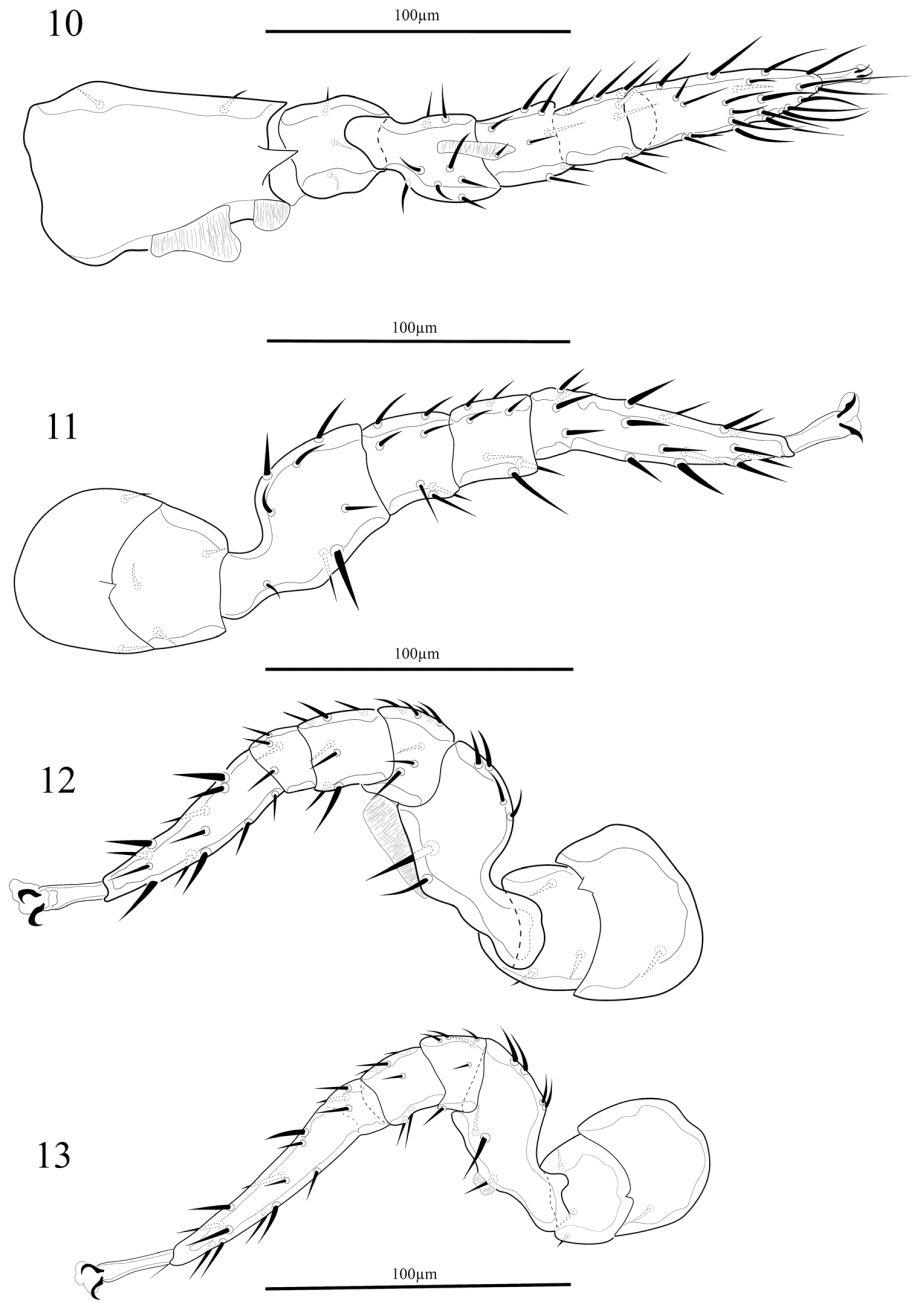
*Oplitis exopodi* (female): **10** Leg I; **11** Leg II; **12** Leg III **13** Leg IV.

*Legs* (Figs 10–13). Leg chaetotaxy agree with Evans, 1972. Leg lengths: leg I 251–263, leg II 240–255, leg III 214–225, and leg IV 224–238 long.

##### Remarks.

*Oplitis exopodi* was originally found by Hunter and Farrier (1975) from North Carolina (USA) in association with *Solenopsis xyloni* and *Brachyponera solitaria* (Formicidae). It can be distinguished from other known species by dorsal shield smooth and with 105 pairs of scimitar-shaped setae; ventral regions (excluding exopodals) smooth; genital shield elongate, ratio length/width = 1.21–1.23; perigenital ring oblong, with 5 crenulations, ratio length/width = 1.69–1.73; peritremes long and three-convoluted; pre-anal line present; *Ad2* shorter than *Ad1*; pedofossae IV narrow and with rounded tip.

Our specimens of *Oplitis exopodi* show some variations with illustration given by Hunter and Farrier (1975), especially shape of anterior section of peritreme (notched in our specimens, but smooth in with Hunter and Farrier’s illustration), shape of pedofossae III (foot-shaped in our specimens, but wide and pointed in Hunter and Farrier’s illustration) and the number of scimitar-shaped setae on ventral region (4-5 pairs in our specimens, but 6-8 pairs in Hunter and Farrier’s illustration).

##### Material examined.

2 females, Iran, Mazandaran Province, Nowshahr, Kheyrood-Kenar forest, 36°34'N, 50°34'E, altitude 741 m a.s.l., 27 May, 2015, E. Babaeian coll., in leaf-litter. The type specimens are deposited in the Acarological Collection, Jalal Afshar Zoological Museum, Department of Plant Protection, Faculty of Agriculture, University of Tehran, Karaj, Iran.

#### Oplitis sarcinulus

Taxon classificationAnimaliaMesostigmataOplitidae

Hunter & Farrier, 1976

Figs 14–21

##### Description.

**Female (n = 9).** *Idiosoma*. Length 473–507, width 400–438.

**Figures 14–21.**
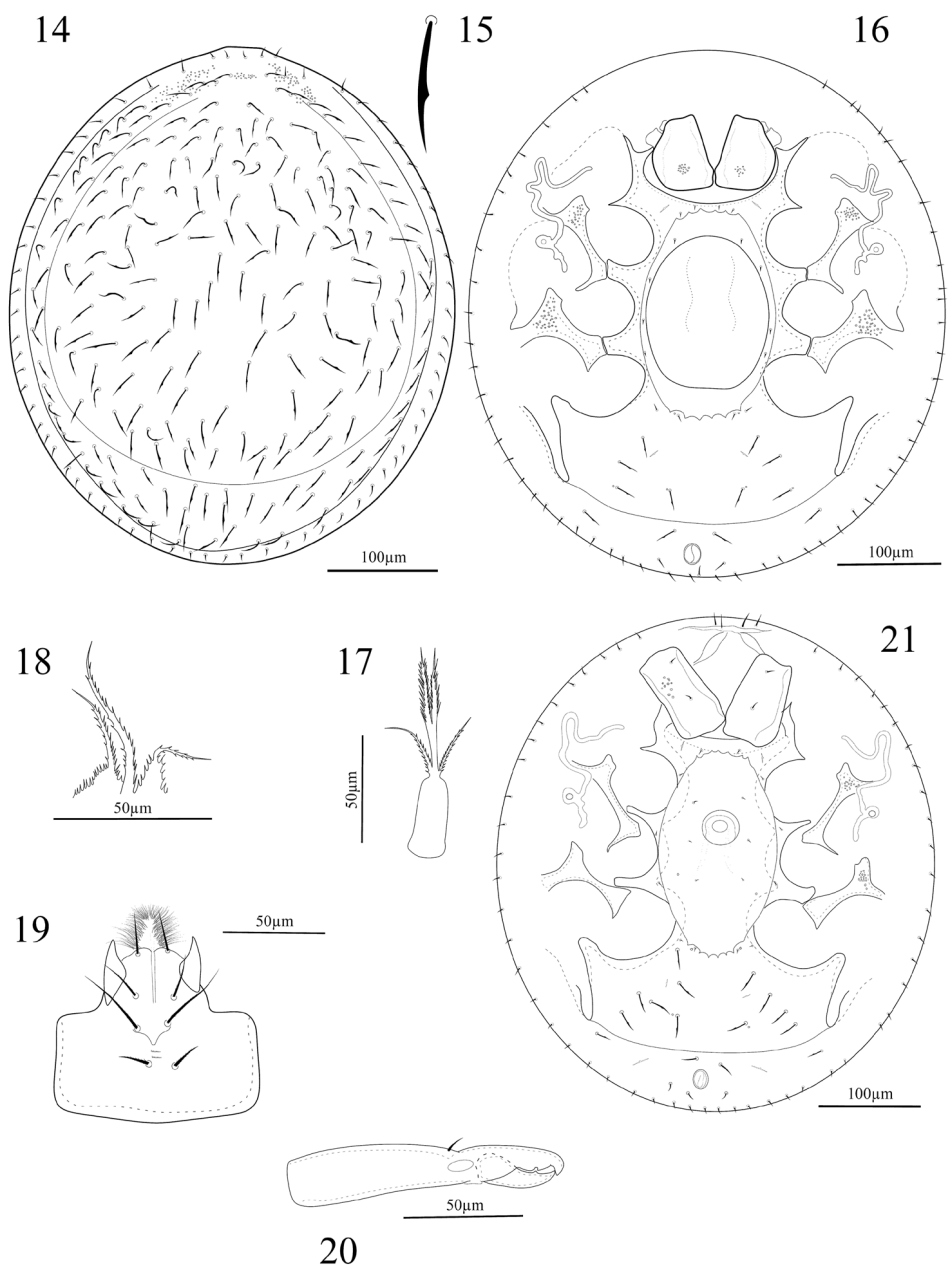
*Oplitis sarcinulus* (female); **14** Dorsal view of idiosoma **15** Dorsal seta **16** Ventral view of idiosoma **17** Tritosternum **18** Epistome **19** Hypostome **20** Chelicera **21** Ventral view of idiosoma (Male).

*Dorsum* (Fig. 14). Dorsal shield oval, smooth on whole surface except on anterior part. Dorso-central region with complement of 106 pairs of scimitar-shaped setae, 25–30 long (Fig. 15). Marginal shield united anteriorly with dorso-central region, with about 29 pairs of smooth and needle-like setae.

*Venter* (Fig. 16). Tritosternal base narrow, lacinia pilose, and three branched, lateral branches shorter than bifurcate median branch (Fig. 17). Sternal region smooth, united with endopodal shields. Genital shield smooth, 135–150 long and 108–128 wide, ratio length/width = 1.20–1.23, situated between coxae II–IV, anterior margin convex and posterior margin straight. Perigenital ring oblong, 175–200 long and 125–145 wide, ratio length/width = 1.40–1.53, extending slightly beyond posterior level of coxae IV, with 5 crenulations on both anterior and posterior margins; lateral margins smooth and continuous. Perigenital area with four pairs of short setae, lateral to genital shield; with two pairs of short setae near anterior margin, one pair near to posterior margin and one pair inserted between coxae IV on inguinal region. Pre-anal line present. Ventral shields with 4–5 pairs of scimitar-shaped setae, adanal setae *Ad1* scimitar-shaped and 20–22 long, *Ad2* smooth, needle-like, and 12–14 long. Peritremes with three subequal convolute branches. Pedofossae II–IV well developed. Pedofossae IV narrowing posteriorly and with a rounded tip.

*Gnathosoma* (Figs 18–20). Epistome three-branched and with serrated margin, median branch longer than laterals (Fig. 18). Corniculi short and horn-like; internal malae numerous and brushy, gnathosomal setae *h1* smooth, *h2* with a few barbs, *h3* and *pc* thickened and denticulated (Fig. 19). Cheliceral digits normal for the genus; movable digit 37 long, with one subdistal tooth and a terminal hook; fixed digit 40 long (from tip to the base of movable digit), with two teeth and a terminal hook; pilus dentilis setiform and minute (Fig. 20). Palptarsus with three-tined apotele. Leg lengths: legs I 245–250, legs II 210–220, legs III 175–188, and legs IV 213–225 long.

**Male (n = 3).** *Idiosoma*. Length 438–450, width 385–395.

*Dorsum*. Dorsal setae, shields and puncta as female.

*Venter* (Fig. 21). Ventral region (excluding exopodal shields) smooth, genital aperture rounded and located between coxae II–III. Perigenital ring 188–200 long, 110–125 wide. Peritreme uni-convoluted, U-shaped in central part and without medial extension. *Legs*. Leg I 215–225, leg II 185–205, leg III 165–179, and leg IV 190–201 long.

##### Remarks.

*Oplitis sarcinulus* was described from North Carolina (USA) by Hunter and Farrier (1976) in association with *Tetramorium caespitum* Linnaeus, and is now recorded in Iran for the first time, from leaf-litter. Herein, we provide the complete description and illustration of this species based on adult female and male. *Oplitis sarcinulus* is similar to *Oplitis leonardiana* (Berlese, 1903) and *Oplitis pennsylvanica* (Berlese, 1903). On the basis of some photomicrographs from three very good quality slides (No. 2/16, 2/17 and 2/19) kindly supplied by Dr. Nannelli (Istituto Sperimentale per la Zoologia Agraria, Florence, Italy), we found that these two species are clearly different from *Oplitis sarcinulus*. It differs from *Oplitis leonardiana* (Berlese, 1903) and *Oplitis pennsylvanica* (Berlese, 1903) by the shape of the peritreme (M-shaped in *Oplitis sarcinulus*, but U-shaped and with a small extension as Fig. 8 in *Oplitis pennsylvanica*, but with this U-shaped bend more compressed, posteriorly curved and directed posteriorly as Fig. 9 in *Oplitis leonardiana*), ventral shield (with 4–5 pairs of setae in *Oplitis sarcinulus* and *Oplitis pennsylvanica*, but 8–9 pairs in *Oplitis leonardiana*), adanal setae *Ad2* (shorter than *Ad1* in *Oplitis sarcinulus*, but as long as *Ad1* in both *Oplitis leonardiana* and *Oplitis pennsylvanica*), shape of genital shield (narrowly oval and elliptical in *Oplitis sarcinulus* and *Oplitis leonardiana*, but widely oval and beehive-shape in *Oplitis pennsylvanica*), width and the number of posterior crenulations (narrow and with 5–6 crenulations in *Oplitis sarcinulus*, but wide and with 9 crenulations in *Oplitis pennsylvanica* and *Oplitis leonardiana*), and pedofossae IV (narrow and with rounded distal end in *Oplitis sarcinulus*, but wide, with pointed and rounded distal end in *Oplitis leonardiana* and *Oplitis pennsylvanica*).

The Iranian specimens agree well with the original description, however, Hunter and Farrier (1976) mentioned 6–7 crenulations on anterior and posterior margins of perigenital ring, respectively, and sternal region of perigenital ring with small punctuation, but with 5–6 crenulations and sternal region smooth in Iranian specimens.

##### Material examined.

6 females and 3 males, Iran, Mazandaran province, Nowshahr, Kheyrood-Kenar forest, 36°34'N, 051°33'E, altitude 636 m a.s.l., 5 July 2014, E. Babaeian coll., in leaf-litter. The type specimens are deposited in the Acarological Collection, Jalal Afshar Zoological Museum, Department of Plant Protection, Faculty of Agriculture, University of Tehran, Karaj, Iran.

### Key to the Iranian species of *Oplitis* (females)

**Table:** 

1	Distance between anterior margin of genital shield to anterior margin of perigenital ring is longer than posterior distance, peritremes mushroom-like	***Oplitis exopodi* Hunter & Farrier, 1975**
–	Distance between anterior margin of genital shield to anterior margin of perigenital ring is equal or shorter than posterior distance, peritremes M- or U-shaped	**2**
2	Dorsal and ventral shield setae smooth and needle-like, peritremes M-shaped and anterior loop of prestigmatic section shorter than posterior one	***Oplitis iranicus* Kazemi & Kontschán, 2007**
–	Dorsal and ventral shield setae scimitar-shaped, anterior loop of prestigmatic section longer than posterior	**3**
3	Prestigmatic part U-shaped, marginal and ventral shields completely with small punctations, ratio length/width of genital shield ≈ 1.45, ratio length/width of perigenital ring ≈ 1.90	***Oplitis paradoxa* (Canestrini & Berlese, 1884)**
–	Peritremes more M-shaped and prestigmatic part longer than posterior part, only anterior part of marginal and dorsal shields with small punctations, ratio length/width of genital shield ≈ 1.20–1.23, ratio length/width of perigenital ring ≈ 1.40–1.53	***Oplitis sarcinulus* Hunter & Farrier, 1976**

## Supplementary Material

XML Treatment for Oplitis

XML Treatment for Oplitis exopodi

XML Treatment for Oplitis sarcinulus
